# Knockdown of YTHDF2 initiates ERS-induced apoptosis and cancer stemness suppression by sustaining GLI2 stability in cervical cancer

**DOI:** 10.1016/j.tranon.2024.101994

**Published:** 2024-05-21

**Authors:** Fujian Wan, Fengwu Qiu, Yang Deng, Hao Hu, Yingjie Zhang, Jia-Yu Zhang, Pei Kuang, Haoyu Tian, Dewang Wu, Hang Min, Jiapeng Li, Jing Xu, Jun Zhou

**Affiliations:** a Institute of Biology and Medicine, College of Life and Health Sciences, Wuhan University of Science and Technology, Wuhan 430081, China; b Hubei Institute of Blood Transfusion, Wuhan Blood Center, No.8 Baofeng Road, Qiaokou District,Wuhan, Hubei 430081, PR China

**Keywords:** Cervical cancer, YTHDF2, Tumor stemness, Apoptosis, ERS, JNK, GLI2

## Abstract

Cervical cancer ranks fourth in women in terms of incidence and mortality. The RNA-binding protein YTH N6-methyladenosine RNA-binding protein F2 (YTHDF2) contributes to cancer progression by incompletely understood mechanisms. We show how YTHDF2 controls the fate of cervical cancer cells and whether YTHDF2 could be a valid target for the therapy of cervical cancer. Sphere formation and alkaline phosphatase staining assays were performed to evaluate tumor stemness of cervical cancer cells following YTHDF2 knockdown. Apoptosis was detected by flow cytometry and TUNEL assay. The compounds 4PBA and SP600125 were used to investigate the correlation between JNK, endoplasmic reticulum stress, tumor stemness, and apoptosis. Data from The Cancer Genome Atlas (TCGA) databases and Gene Expression Omnibus (GEO) revealed that GLI family zinc finger 2 (GLI2) might be the target of YTHDF2. The transcription inhibitor actinomycin D and dual-luciferase reporter gene assays were employed to investigate the association between the GLI2 mRNA and YTHDF2. Nude mouse xenografts were generated to assess the effects of YTHDF2 knockdown on cervical cancer growth in vivo. Knockdown of YTHDF2 up-regulated the expression of GLI2, leading to JNK phosphorylation and endoplasmic reticulum stress. These processes inhibited the proliferation of cervical cancer cells and their tumor cell stemness and promotion of apoptosis. In conclusion, the knockdown of YTHDF2 significantly affects the progression of cervical cancer cells, making it a potential target for treating cervical cancer.

## Highlights

• YTHDF2 knockdown suppresses the growth of cervical cancer cells and tumor stemness while also promoting apoptosis.
• Induction of endoplasmic reticulum stress is the cause of YTHDF2 knockdown-induced suppression of tumor stemness and promotion of apoptosis.
• The activation of the JNK pathway through up-regulation of GLI2 expression is responsible for YTHDF2 knockdown-induced promotion of endoplasmic reticulum stress.
• YTHDF2 regulates the expression of GLI2 by targeting the 3′UTR of GLI2 mRNA. Additionally, phosphorylated JNK promotes the upregulation of GLI2 expression.

## Introduction

Cervical cancer is the fourth most common and deadly cancer in women [1], with its incidence in lactating women second only to that of breast cancer [2]. Approximately 604,127 new cases of cervical cancer and 341,831 cervical cancer-related deaths are expected worldwide by 2020 [3]. Cancer poses a significant threat to women's health and life. The etiology of cervical cancer is multifactorial and involves HPV infection, tobacco use, immunosuppression, and prolonged oral contraceptive use [4]. HPV vaccination is a promising strategy for preventing HPV-mediated cervical cancer [5,6]. However, vaccination rates remain low in middle- and low-income countries, with approximately 80 % of new global cervical cancer cases arising in these regions [7]. The vaccines do not provide immunity against all HPV strains. Therefore, routine screening for cervical cancer is crucial for the early detection of signs of malignancy in the cervix [8]. Traditional treatments, including surgery, radiotherapy, and chemotherapy, have demonstrated effectiveness only for early stage cervical cancer [9]. Therefore, it is imperative to explore novel mechanisms, targets, and techniques to enhance cervical cancer prognosis and achieve a five-year survival rate.

N6-methyladenosine (m6A) is the most common internal transcriptome modification in eukaryotes, which involves the addition of a methyl group to the sixth nitrogen atom of adenine. It plays a vital role in regulating gene expression [10]. YTH N6-methyladenosine RNA-binding protein F2 (YTHDF2) is an m6A "reader" that can decrease RNA stability by binding to the m6A site [11]. YTHDF2 has been reported to participate in numerous biological processes [12,13]. YTHDF2 affects the stemness of multiple cancer cells [[14], [15], [16], [17]]. It affects cancer progression by regulating mRNA degradation in various cancers [18]. Additionally, it inhibits the epithelial–mesenchymal transition and enhances the sensitivity of cervical cancer cells to drugs by targeting AXIN1 [19]. Numerous reports have shown that YTHDF2 knockdown can induce apoptosis in multiple cancers [[20], [21], [22]]. However, the effect of YTHDF2 on cervical cancer cell stemness has not been studied. The aim of this study was to explore whether YTHDF2 could be a therapeutic target for cervical cancer cells and to determine the possible mechanism of action to provide new therapeutic strategies for the treatment of cervical cancer.

GLI family zinc finger 2 (GLI2) belongs to the C2H2-type zinc finger protein subclass of the Gli family [23]. Constitutive Hedgehog/GLI2 signaling can drive extracutaneous basal-like squamous cell carcinoma progression and bone remodeling [24]. Additionally, GLI2 contributes to poor clinical outcomes in patients with oral squamous cell carcinoma [25]. These studies suggest that GLI2 can be involved in cancer development.

The endoplasmic reticulum (ER) is an intracellular organelle primarily responsible for the synthesis, maturation, and modification of post-translated or co-translated secreted proteins and membrane proteins, and plays a role in various metabolic processes [26]. Disruption of ER homeostasis can lead to ER stress (ERS), which has a variety of complex causes, including disruption of intracellular calcium homeostasis, DNA damage, and alterations in redox status [27]. ERS triggers a complex signaling process known as the unfolded protein response (UPR). ERS and the UPR mediate various molecular and biochemical processes that affect cell proliferation, differentiation, and programmed cell death [28,29]. Notably, YTHDF2 knockdown has been shown to promote ERS in MYC-induced triple-negative breast cancers, resulting in cytotoxicity and apoptosis [30].

This study examined the correlation between YTHDF2 expression, cervical cancer stemness, and apoptosis. These results indicate that YTHDF2 knockdown enhanced apoptosis and suppressed tumor stemness in cervical cancer cells. Subsequent analysis verified this effect through the promotion of ERS. Further verification demonstrated that YTHDF2 functions through GLI2 regulation to influence ERS.

## Materials and methods

### Data collection

All data used in this study were downloaded from the TCGA and GEO databases. The RNAseq data from the STAR process of the TCGA-CESC (cervical endocervical adenocarcinoma and squamous cell carcinoma) project were downloaded and organized, the data in TPM format were extracted, and the data downloaded from the GEO database were analyzed and organized using the R language (version 4.2.2) for analysis and organization.

### Cell culture

Human cervical normal cells HCerEpiC (WN-10,188) and human cervical cancer cells C-33A (WN-10,252), SiHa (WN-10,125), and CaSki (WN-10,172) were purchased from Warner Biotechnology, and HeLa (BNCC342189) cells were purchased from BeiNa Culture Collection. HCerEpiC, C-33A, and SiHa cells were cultured in MEM (GIBCO) containing 10 % serum (GIBCO); HeLa cells were cultured in DMEM (GIBCO) containing 10 % serum (GIBCO); and Ca Ski cells were cultured in RPMI 1640 medium (GIBCO) containing 10 % serum (GIBCO). Cells were cultured in a constant-temperature incubator at 37 °C with 5 % CO_2_.

### Transfection

shYTHDF2 and the interference control were synthesized by Beijing Tsingke Biotech, and siGLI2 and the interference control were synthesized by Sangon Biotech (Shanghai), as listed in Supplementary Table 1. HeLa and SiHa cells stably expressing shYTHDF2 were obtained by lentiviral packaging of the infected cells and screened with puromycin for 4 weeks. siRNA was used to infect cells using Lipofectamine 2000 Reagent (ThermoFisher).

### qPCR

When the cells reached 80 % confluence, they were harvested and lysed with TRIZON, followed by extraction of total RNA using an RNA Extraction Kit (Cat. No. AG21017; Accurate Biology), according to the manufacturer's instructions, and reverse transcription was performed using the ABScript II cDNA First-Strand Synthesis Kit (Cat. No. RK20400; Abclonal Technology) according to the manufacturer's instructions for obtaining cDNA. The obtained cDNA was mixed with SYBR Green (Cat. No. 11201ES03; Shanghai Yeasen Biotechnology) at a specific ratio, and the final cq value was monitored using a PCR instrument (Bio-Rad Laboratories), which was repeated three times per tube. The results were analyzed using the BioRad CFXManager software. The qPCR primer sequences are listed in Supplementary Table 2.

### Western blotting (WB)

After 80 % confluence was reached, the cells were harvested, and complete lysis was achieved using RIPA cell lysis solution (MeilunBio). Cell remnants were precipitated by centrifugation at 12,000 *g* for 15 min. The supernatant was collected, and the concentration of total proteins was measured using a BCA Protein Quantification Kit (MeilunBio). The proteins were diluted to the appropriate concentration and mixed with the loading buffer at an appropriate ratio. They were then subjected to heating in a metal bath for 10 min, applied equally to SDS-PAGE for separation. Following the electrophoresis, the target proteins were separated on a gel and transferred onto a 0.44-μm PVDF membrane using a membrane transfer apparatus. After a one-hour milk or BSA blocking step at room temperature, the membrane was incubated overnight at 4 °C with antibody dilutions, followed by a 1-h incubation at room temperature with secondary antibody dilutions, and then imaged under a chemiluminescence imager after incubation with an ECL chemiluminescence kit and quantified by densitometry using the ImageJ software (NIH, Bethesda, USA). At least three independent experiments were carried out, and representative images are shown. Information on all antibodies used in this experiment is provided in Supplementary Table 3.

### Colony formation

After the cells were digested with trypsin while they were in a good growth state, the digestion was stopped with serum-containing medium 1:1 to obtain a cell suspension, which was then centrifuged at 300 *g* for 5 min. The supernatant was removed, and the cells were resuspended in complete medium, counted, and then seeded into 6-well plates after being diluted to 1000 cells/2 ml of complete medium. The cells were then incubated in a 37 °C incubator with 5 % CO_2_. The medium was replaced every day. After 7 days, the culture was terminated, and the cells were stained with 0.1 % crystal violet for 15 min. The remaining crystal violet was removed using fresh water and dried for imaging. At least three independent experiments were carried out, and representative images are shown.

### EdU assay

Under optimal growth conditions, cells were seeded into 24-well plates with a circular microscope cover glass (Wuxi NEST Biotechnology) attached at a density of 50,000 cells per well and incubated overnight at 37 °C in a cell culture incubator containing 5 % CO_2_. Cells on a circular microscope cover glass were stained using an EdU kit (Cat. No. MA0425/MA0424 Dalian Meilun Biology Technology) according to the manufacturer's instructions and imaged under a fluorescence microscope after staining. At least three independent experiments were carried out, and representative images are shown.

### Cell counting kit-8

Well-grown cells were inoculated into 96-well plates in sets of four replicates of 5000 per well. The plates were cultured in an incubator until the cells adhered to the walls. One of the 96-well plates was removed and subjected to complete medium supplemented with 10 % CCK8 working solution (Abclonal Technology, Cat. No. RM02823). The plate was then returned to the incubator and incubated for 2 h, followed by measurement of absorbance at 450 nm using a multifunctional enzyme-labeling instrument. Absorbance was initially measured at 450 nm, followed by repeated measurements at 24, 48, and 72 h to record the absorbance at the same wavelength.

### Sphere formation

When the cells were in the logarithmic growth phase, they were digested and resuspended using trypsin, and the digestion was terminated using serum-containing medium. The supernatant was removed by centrifugation. The cells were resuspended and counted using DMEM/F12 (Melan) medium containing 1 % B27 (Gibco), 20 ng/mL EGF, and 20 ng/mL bFGF (T&L Biological Technology), and 10,000 cells were collected and inoculated into low-adsorbent 24-well plates (Corning). Incubation was continued for 7 days, during which time the single cell wells were recorded. The 24-well plates were placed in an inverted microscope and imaged on the seventh day. At least three independent experiments were carried out, and representative images are shown.

### Ap staining assay

When the cells reached optimal growth, they were digested with trypsin, and the digestion was halted with medium to obtain the cell suspension. This cell suspension was then seeded with a specific number of cells in a 6-well plate, cultured overnight in a cell culture incubator, and subsequently fixed with 4 % formaldehyde fixative. Following fixation, the cells were processed using an alkaline phosphatase staining kit (Yeasen Biotechnology) Staining procedures followed the manufacturer's instructions. Excess staining solution was removed and the cells were imaged under an inverted fluorescence microscope. At least three independent experiments were carried out, and representative images are shown.

### PI\Annexin V staining

Cells in the logarithmic growth phase were harvested and treated with ethylenediaminetetraacetic acid-free trypsin. The digestion reaction was halted using complete medium, and the cells were washed thrice with pre-cooled PBS containing 5 % FBS and centrifuged at 300 *g* for 5 min to remove the supernatant. The cells were stained using a propidium iodide (PI)/annexin V staining kit (Cat. No. 40311ES20; Shanghai Yeasen Biotechnology) according to the manufacturer's instructions. Apoptosis was analyzed using flow cytometry. At least three independent experiments were carried out and representative images are shown.

### TUNEL assay

Cells were seeded at approximately 50 % confluence on a circle microscope cover glass when in a good growth state, and incubation was stopped after 24 h in a 37 °C incubator containing 5 % CO_2_. Subsequently the cells were fixed and stained of the cells using a One Step TUNEL Apoptosis Assay Kit (Cat. No. C1089; Beyotime Biotechnology) following the manufacturer's instructions. After staining, imaging was performed using an inverted fluorescence microscope. At least three independent experiments were carried out and representative images are shown.

### Lipid peroxidation assessment

Cells in a well-growing state were inoculated into six-well plates at 50 % confluence and incubated in a constant temperature incubator at 37 °C with 5 % CO_2_ until they adhered to the wall. Then a certain concentration of C11 BODIPY 581/591 was added to reach a final concentration of 10 μm. After cells were incubated for 1 h in the cell incubator, excess dye was washed off with PBS. The cells were then digested using trypsin, followed by resuspension in PBS with 5 % FBS. After being centrifuged at 300 *g* for 5 min to remove the supernatant, the cells were resuspended in PBS containing 5 % FBS. Subsequently, cellular oxidative stress was analyzed using a flow cytometer.

### GO, KEGG analysis, and GSEA

Differential expression analysis was performed using the R package DEseq2 (R version 4.2.2), whereas GO, KEGG, and GSEA analysis were conducted using the R package clusterProfiler. The final outcomes were visualized using the ggplot2 package.

### RNA stability assay

Well-cultured cells were treated with actinomycin D for 0, 2, and 6 h, and RNA was extracted. The half-life of GLI2 was determined using a previously described quantitative qPCR method.

### Dual luciferase reporter assay

Cells were seeded in 24-well plates, transfected with plasmids of the dual-luciferase reporter gene using Lipofectamine 2000 Reagent (ThermoFisher). After 24 h, the cells were lysed, and the cell lysates were diluted to a specific ratio and added to the luciferase substrate using a Firefly & Renilla Luciferase Reporter Assay Kit (Catalog No. MA0518–1; Dalian Meilun Biology Technology) according to the manufacturer's instructions. Chemiluminescence was measured using a multifunctional enzyme-labeling instrument.

### Xenograft generation

Two groups of five female BALB/c-nu mice, aged 3–4 weeks, were randomly assigned to each cage. Mice in both groups were subcutaneously injected with 2 × 10^6^ cell suspension in PBS. One group was injected with shYTHDF2, and the other group was injected with sh-NC. The volume of the transplanted tumors was recorded at 3-day intervals until 7 days after feeding. Mice were euthanized after a specific period. The experiments were conducted between July and August 2023 in an SPF-grade laboratory animal facility. Prior to the experiment, the Institutional Animal Care and Use Committee (IACUC) of Wuhan University of Science and Technology (WUST) reviewed the experimental procedures. Animal welfare was properly maintained throughout the experiments.

### Statistical analysis

R (version 4.2.2) and GraphPad (version 9.5.1) were used for all data organization and analysis. All experiments were performed at least three times, and the results were subjected to one-way analysis of variance (ANOVA) or Student's *t*-tests. *p* < 0.05 was considered statistically significant (*p* < 0.05 = *, *p* < 0.01 = **, *p* < 0.001 = ***).

## Results

### YTHDF2 is highly expressed in cervical cancer cell lines and inhibits the progression of cervical cancer after knockdown

**Fig. 1:**
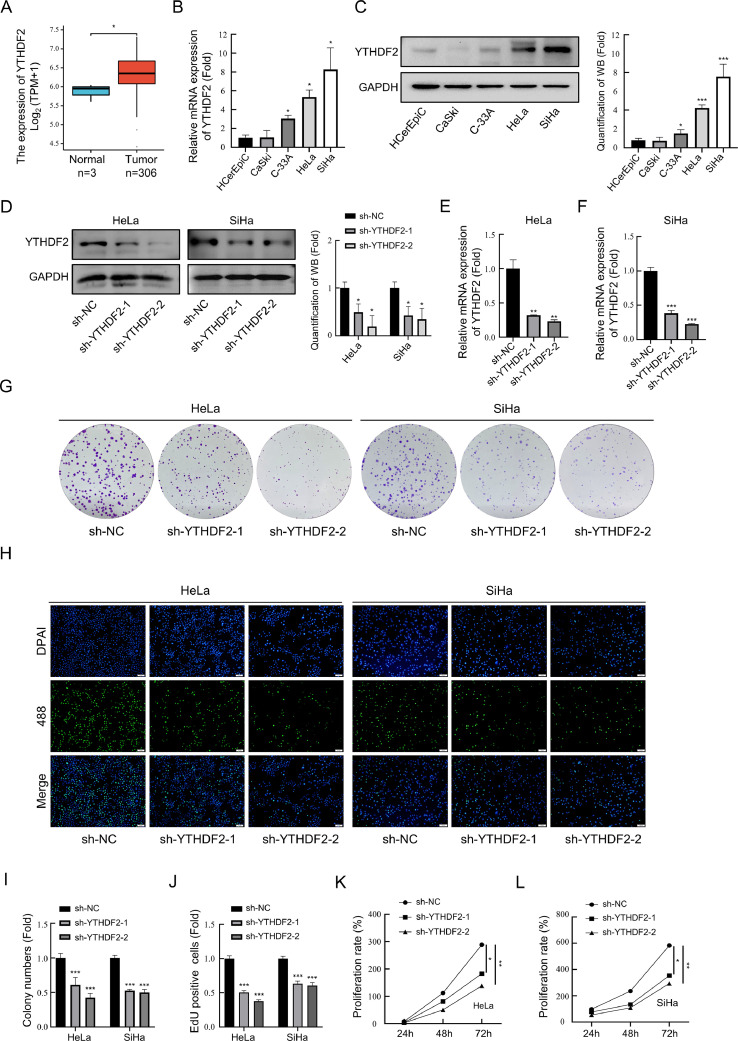
YTHDF2 is highly expressed in cervical cancer cell lines and inhibits the progression of cervical cancer after knockdown. (A) Differential expression analysis of YTHDF2 in cervical cancer based on data from TCGA database. (B) The expression of YTHDF2 evaluated using qPCR in human normal cervical epithelial cell HCerEpiC and cervical cancer cell lines. (C) Representative Western blots of YTHDF2 and their quantitative analysis in HCerEpiC and cervical cancer cell lines. (D, E) Knockdown efficiency of YTHDF2 in HeLa and SiHa cells detected by qPCR. (F) Representative Western blots of YTHDF2 and their quantitative analysis showing knockdown efficiency in HeLa and SiHa cells. (G) Representative images of Colony formation assay performed in control and YTHDF2 stable knockdown HeLa and SiHa cells. (H) Representative images of EdU assay performed in control and YTHDF2 stable knockdown HeLa and SiHa cells. (I) Quantification of colony numbers in control and YTHDF2 stable knockdown HeLa and SiHa cells. (J) Quantification of EdU-positive cells in control and YTHDF2 stable knockdown HeLa and SiHa cells. (K, L) CCK-8 assay performed in YTHDF2 stable knockdown HeLa and SiHa cells. The data represent the mean ± *S*.D. of three independent experiments. ANOVA or *t*-tests was used for statistical analysis. * *p* < 0.05, ** *p* < 0.01, *** *p* < 0.001 indicate a significant difference between the indicated groups.

Analysis of the expression data of cervical cancer in the TCGA database revealed that YTHDF2 expression was up-regulated in cervical cancer tissues compared with that in normal tissues (Fig. 1A). Subsequently, analysis was conducted on the mRNA and protein expression levels of YTHDF2 in the human cervical epithelial cell line HCerEpiC and in the cervical cancer cell lines Ca Ski, C-33A, HeLa, and SiHa. The results revealed a significant up-regulation of YTHDF2 expression in HeLa and SiHa cells compared with that in HCerEpiCs (Figs. 1B and 1C). To investigate this mechanism, stable-transformed cell lines with YTHDF2 knockdown were constructed in HeLa and SiHa cell lines (Figs. 1D–1F). The effect of YTHDF2 knockdown on cervical cancer cell proliferation was assessed using clone formation and EdU assays (Figs. 1G and 1H). Quantifications of the number of clones and EdU-positive cells are presented in Figs. 1I and 1J, respectively. The effect of YTHDF2 knockdown on cervical cancer cell viability was evaluated using the CCK8 assay (Figs. 1K and 1L). These results demonstrate that YTHDF2 knockdown impedes the proliferation and viability of cervical cancer cells.

### YTHDF2 knockdown suppresses tumor stemness in cervical cancer cells

**Fig. 2:**
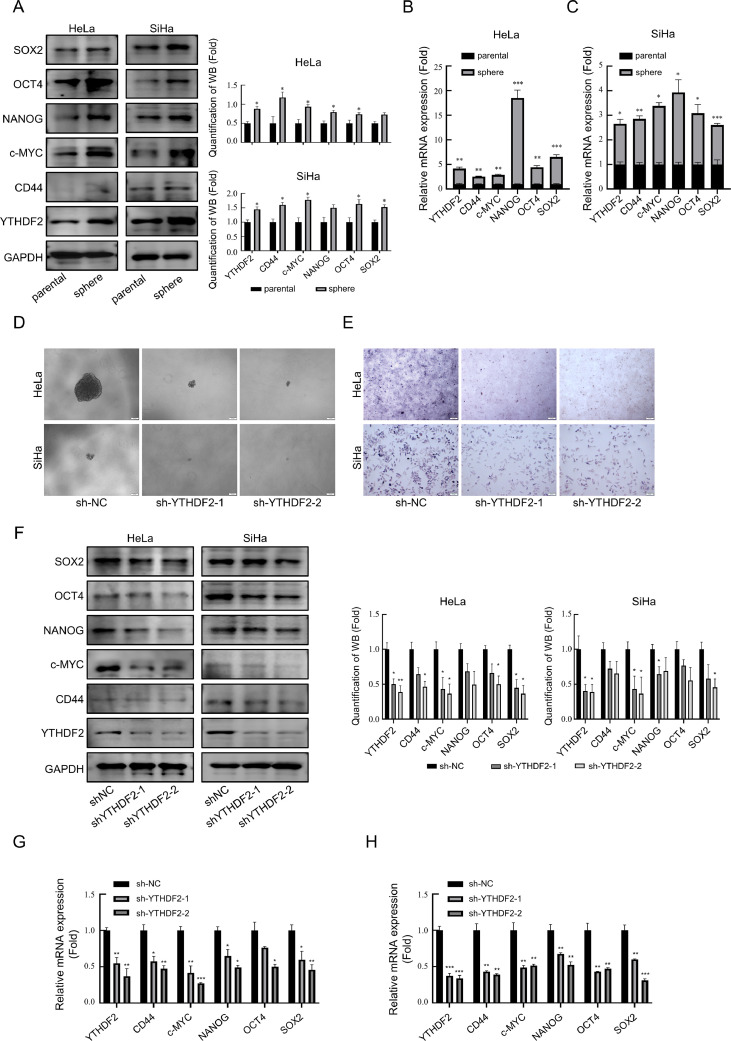
YTHDF2 knockdown suppresses tumor stemness in cervical cancer cells. (A) Representative Western blots of stemness-related genes and YTHDF2, as well as their quantitative analysis in parental and suspension-cultured HeLa and SiHa cells. (B, C) mRNA expression of stemness-related genes and YTHDF2 detected by qPCR after suspension culture of HeLa and SiHa cells. (D) Representative images of sphere formation assay performed in YTHDF2 stable knockdown HeLa and SiHa cells. (E) Representative images of AP staining assay performed in YTHDF2 stable knockdown HeLa and SiHa cells. (F) Representative Western blots of stemness-related genes and their quantitative analysis in YTHDF2 stable knockdown HeLa and SiHa cells. (G, H) Expression of stemness-related genes was detected by qPCR in HeLa and SiHa cells after knockdown of YTHDF2. The data represent the mean ± SD of three independent experiments. ANOVA or *t*-tests were used for statistical analysis. * *p* < 0.05, ** *p* < 0.01, *** *p* < 0.001 indicate a significant difference between the indicated groups.

Numerous reports have suggested that YTHDF2 can impact cancer progression by affecting the tumor stemness during carcinogenesis [[31], [32], [33]]. Subsequently, the relationship between the expression level of YTHDF2 and the tumor stemness of cervical cancer cells was evaluated. Cervical cancer cell lines exhibiting enhanced stemness were obtained by suspension culture of cervical cancer cells. Subsequently, the differences in mRNA and protein expression of stemness-related genes, as well as YTHDF2, between the suspension-cultured cervical cancer cell lines and their parental cell lines adherently cultured in cell culture plates were compared (Figs. 2A–2C). The study findings revealed that enhanced stemness in cervical cancer cells was achieved by up-regulating the expression of stemness-related genes in suspension culture. Subsequently, both the mRNA and protein levels of YTHDF2 were further elevated in the stemness-enhanced cells. Moreover, YTHDF2 expression correlated positively with cell stemness in enhanced cells. Additionally, the size of the suspension cell spheres was significantly reduced in YTHDF2 knockdown cells cultured in suspension compared with sh-NC cells (Fig. 2D). Additionally, alkaline phosphatase expression level was significantly reduced upon YTHDF2 knockdown, as shown by AP staining (Fig. 2E). After analyzing the expression levels of stemness-related genes following the knockdown of YTHDF2, as shown in Figs. 2F–2H, it was discovered that there was a specific degree of reduction in their expression due to YTHDF2 knockdown. These findings indicated that YTHDF2 expression affects cervical cancer stemness.

### Knockdown of YTHDF2 promoted apoptosis in cervical cancer cells

**Fig. 3:**
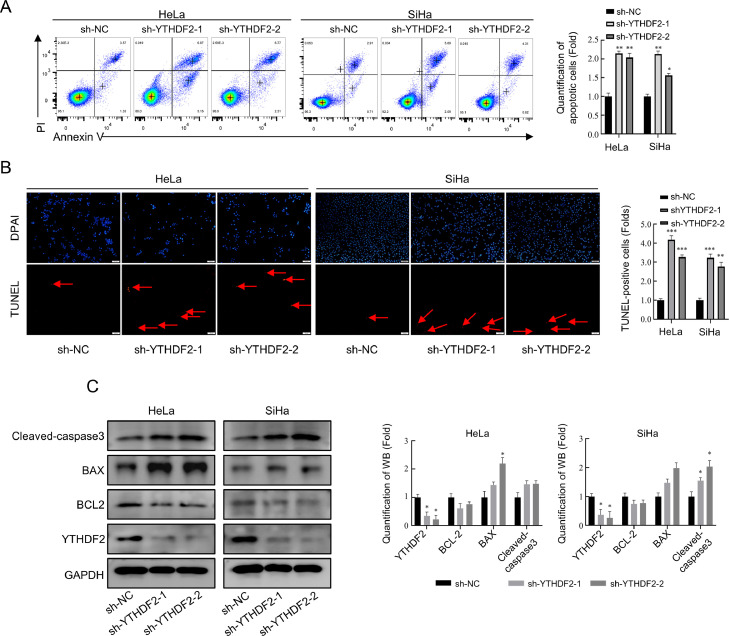
Promotion of apoptosis in cervical cancer cells after knockdown of YTHDF2. (A) Representative images of flow cytometry and their quantitative analysis performed in YTHDF2 stable knockdown HeLa and SiHa cells showing the percentages of PI and Annexin V double positive cells. (B) Representative images of TUNEL assay and their quantitative analysis performed in YTHDF2 stable knockdown HeLa and SiHa cells. (C) Representative Western blots of apoptosis-related genes and YTHDF2, as well as their quantitative analysis in YTHDF2 stable knockdown HeLa and SiHa cells. The data represent the mean ± SD of three independent experiments. ANOVA or *t*-tests were used for statistical analysis. * *p* < 0.05, ** *p* < 0.01, *** *p* < 0.001 indicate a significant difference between the indicated groups.

The knockdown of YTHDF2 resulted in significant inhibition of sphere formation ability, according to the tumor sphere formation assay. However, there was no significant reduction in stemness-related genes based on protein and mRNA expression analysis. Further investigation is necessary to determine the collective influence of other factors on tumor sphere formation ability in cervical cancer. YTHDF2 knockdown induces apoptosis in multiple cancers [20,34,35]. Therefore, we investigated the effect of apoptosis in cervical cancer cells after YTHDF2 knockdown. The percentage of apoptotic cells significantly increased after YTHDF2 knockdown, as evidenced by PI/Annexin V staining and subsequent flow cytometric analysis (Fig. 3A). This effect was further confirmed by TUNEL assay (Fig. 3B) and WB analysis (Fig. 3C). Data on apoptosis-related protein expression levels indicated that cervical cancer cells underwent apoptosis following YTHDF2 knockdown. These findings suggest that the knockdown of YTHDF2 could also lead to a certain degree of apoptosis in cervical cancer cells.

### Homeostatic dysregulation of YTHDF2 may be associated with RNA metabolism, protein metabolism, and cellular stress

**Fig. 4:**
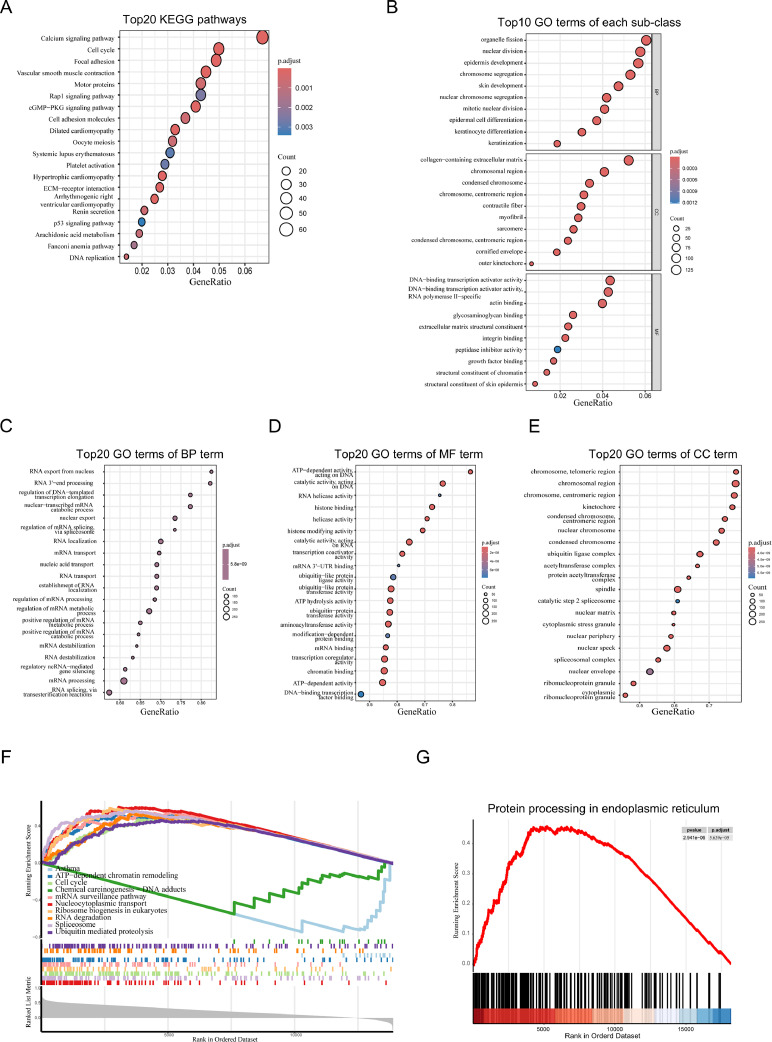
Homeostatic dysregulation of YTHDF2 may be associated with RNA metabolism, protein metabolism and cellular stress. (A, B) KEGG and GO analysis were performed on CESC samples in the TCGA database. (C–F) gseGO and gseKEGG analysis of genes correlated with the expression of YTHDF2 of CESC in TCGA database. (G) gseKEGG analysis of genes correlated with the expression of YTHDF2 in TCGA cervical adenocarcinoma database.

To analyze the factors that influence the stemness of cervical cancer cells, as well as apoptosis after YTHDF2 knockdown, the expression data of genes in cervical cancer in TCGA data were compiled and a series of analyses were performed. KEGG analysis of differentially expressed genes between cervical cancer and tumors in the TCGA database indicated that many enriched pathways were related to cell growth. This suggests that activation of growth-related pathways is likely the primary cause of cervical cell carcinomas. This finding highlights the main cause of most cellular carcinogens and suggests that the analysis results are reliable (Figs. 4A and 4B). Next, genes that correlated with the expression of YTHDF2 were selected for gseGO and gseKEGG analysis (Figs. 4C–4G). The results revealed a significant association between YTHDF2 and cytoplasmic stress-related pathways pertaining to RNA and protein metabolism. Subsequently, the TCGA cervical adenocarcinoma database was organized and genes with relevance to YTHDF2 were extracted for gseKEGG analysis, and the protein processing in the ER pathway was enriched. The results of these analyses are presented in Data S1. These findings suggest that the homeostasis of YTHDF2 is correlated with the homeostasis of RNA and protein metabolism in cells.

### Knockdown of YTHDF2 leads to oxidative stress and ERS thereby suppressing tumor stemness of cells

**Fig. 5:**
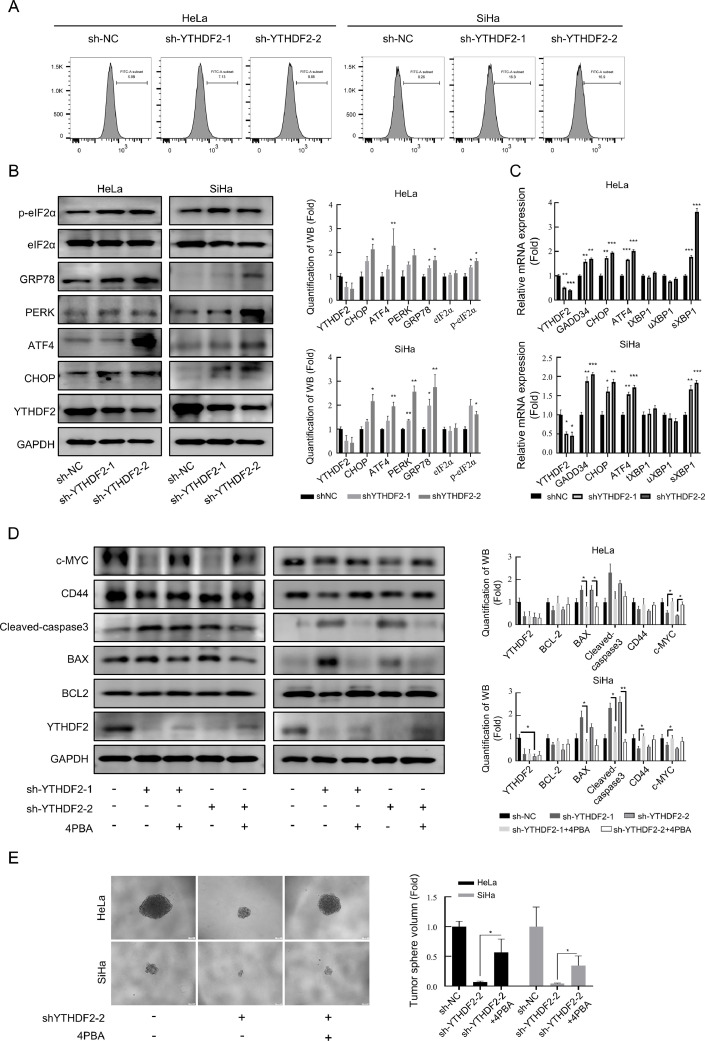
Knockdown of YTHDF2 leads to oxidative stress and ERS, thereby suppressing tumor stemness of cells. (A) Representative images showing levels of cellular reactive oxygen species detected using C11 BODIPY 581/591 by flow cytometry. (B) Representative Western blots of proteins related to ERS and their quantitative analysis in YTHDF2 stable knockdown HeLa and SiHa cells. (C) qPCR was performed to measure the expression of genes related to ERS in YTHDF2 stable knockdown HeLa and SiHa cells. (D) YTHDF2 stable knockdown HeLa and SiHa cells were treated with 4PBA (2 mM) for 24 h, followed by Western blotting. Representative Western blots of genes related to tumor stemness and apoptosis, as well as their quantitative analysis. (E) Representative images of sphere formation assay and their quantitative analysis showing effect on sphere formation ability after introduction of 4PBA (2 mM) in YTHDF2 stable knockdown HeLa and SiHa cells. The data represent the mean ± SD of three independent experiments. ANOVA or *t*-tests were used for statistical analysis. * *p* < 0.05, ** *p* < 0.01, *** *p* < 0.001 indicate a significant difference between the indicated groups.

As disorders of RNA and protein metabolism lead to cellular stress [36,37], the effect of YTHDF2 knockdown on oxidative stress was analyzed (Fig. 5A). The results showed that knockdown of YTHDF2 led to an increase in the level of oxidative stress in cervical cancer cells. Additionally, because the protein processing in the ER pathway was enriched in the gesKEGG analysis of TCGA cervical adenocarcinoma data, ERS has been reported to occur along with ROS production [38,39]. It has been reported that YTHDF2 functions as an m6A reader that binds and degrades mRNAs with m6A sites [11,18]. Therefore, knockdown of YTHDF2 is suspected to lead to the inability of excess mRNA to be degraded in time, resulting in excess translated proteins not being folded in time, leading to ERS. Further examination was performed on the proteins and mRNA expression levels of ER-associated markers (Figs. 5B and 5C). The results demonstrated that the knockdown of YTHDF2 led to an increase in ERS-associated protein and mRNA expression. This suggests that the YTHDF2 knockdown promotes ERS. To evaluate whether the decrease in tumor stemness and the increase in the proportion of apoptotic cells caused by YTHDF2 knockdown were related to an increase in the level of ERS, the ERS-inhibitor 4PBA was used to investigate this relationship. Fig. 5D showed that the addition of 4PBA effectively reversed the decrease in tumor stemness-associated proteins and changes in the expression levels of apoptotic proteins caused by YTHDF2 knockdown. 4PBA also reversed the decrease in the sphere formation ability of cervical cancer cells caused by the knockdown of YTHDF2 (Fig. 5E).

### Knockdown of YTHDF2 induces ERS through the JNK pathway

**Fig. 6:**
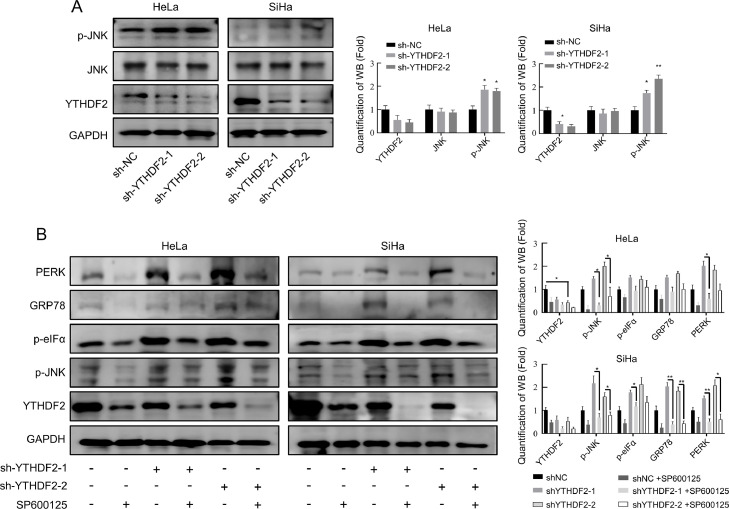
Knockdown of YTHDF2 induces ERS through JNK pathway. (A) Representative Western blots of JNK and p-JNK and their quantitative analysis in YTHDF2 stable knockdown HeLa and SiHa cells. (B) Control and YTHDF2 stable knockdown HeLa and SiHa cells were treated with SP600125 (2 μM) for 24 h. Representative Western blots of p-JNK and ERS-related genes and their quantitative analysis were shown. The data represent the mean ± SD of three independent experiments. *t*-tests were used for statistical analysis. * *p* < 0.05, ** *p* < 0.01 indicates a significant difference between the indicated groups.

The activation of the JNK pathway during ERS in cervical cancer cell lines has been reported previously [40]. It was necessary to verify whether JNK activation occurs through phosphorylation during ERS induced by YTHDF2 knockdown. These findings revealed that YTHDF2 knockdown activated the JNK pathway (Fig. 6A). The addition of SP600125, a JNK inhibitor, significantly reduced ERS-related protein expression (Fig. 6B). These findings suggest that YTHDF2 knockdown promotes ERS by activating the JNK signaling pathway.

### YTHDF2 konckdown activates the JNK pathway through regulation of GLI2, leading to ERS

**Fig. 7:**
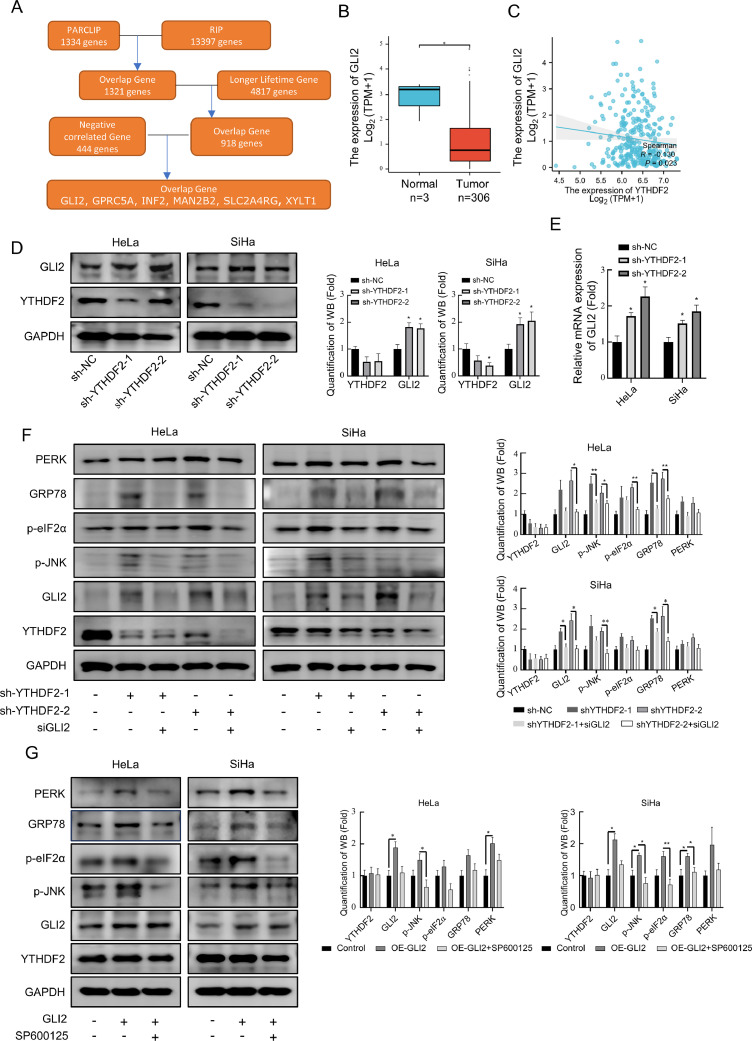
YTHDF2 konckdown activates the JNK pathway through regulation of GLI2, leading to ERS. (A) Workflow diagram for YTHDF2 downstream target gene identification. (B) Differential expression analysis of GLI2 in cervical cancer based on data from TCGA database. (C) Correlation analysis between YTHDF2 and GLI2 in cervical cancer. Data obtained from the TCGA database. (D) Representative Western blots of YTHDF2 and GLI2 and their quantitative analysis in YTHDF2 stable knockdown HeLa and SiHa cells. (E) mRNA expression of YTHDF2 and GLI2 measured by qPCR in YTHDF2 stable knockdown HeLa and SiHa cells. (F) siRNA targeting GLI2 or scramble control was introduced into YTHDF2 stable knockdown HeLa and SiHa cells. Representative Western blots of YTHDF2, p-JNK, and ERS-related genes and their quantitative analysis. (G) HeLa and SiHa cells were transferred with GLI2 or vector for 48 h; SP600125 (2 μM) was introduced into GLI2 transferred group for additional 24 h. Representative Western blots of YTHDF2, p-JNK, and ERS-related genes and their quantitative analysis. The data represent the mean ± SD of three independent experiments. ANOVA or *t*-tests were used for statistical analysis. * *p* < 0.05, ** *p* < 0.01 indicate a significant difference between the indicated groups.

It has been reported that YTHDF2 acts as an m6A reader to degrade mRNAs [11,18]. Whether ERS occurs due to the up-regulation of downstream genes of YTHDF2 after YTHDF2 knockdown remains to be investigated. To identify the target genes, PARCLIP, RIP, and mRNA lifetime data of YTHDF2 knockdown in HeLa cells were downloaded from the GEO database and analyzed, as shown in Fig. 7A. The gene set was obtained by overlapping the PARCLIP and RIP data with the genes with prolonged RNA half-life in HeLa cells after silencing YTHDF2, and finally overlapping with the genes negatively correlated with the expression of YTHDF2 in the cervical cancer expression data in the TCGA database [11]. Six genes, including GLI2, were obtained. The genes that overlapped at each step are listed in Data S2. Analysis of these six genes revealed that only GLI2 was highly expressed in cervical cancer compared with normal tissues in the TCGA database, and it was negatively correlated with the expression of YTHDF2 (Figs. 7B and 7C). Figs. 7D and 7E show that the knockdown of YTHDF2 up-regulated the expression of GLI2. By further silencing the expression of GLI2 in cell lines in which YTHDF2 was knocked down, it was found that silencing the expression of GLI2 by siRNA could effectively reverse the changes in ERS proteins caused by the knockdown of YTHDF2 (Fig. 7F) and that SP600125 was also able to reverse the changes in endoplasmic reticulum stress-related proteins caused by GLI2 (Fig. 7G). These results suggest that the knockdown of YTHDF2 activates the JNK pathway by up-regulating the expression of GLI2, thereby inducing ER stress.

### Knockdown of YTHDF2 up-regulates GLI2 expression by extending the half-life of GLI2 mRNA and activating JNK

**Fig. 8:**
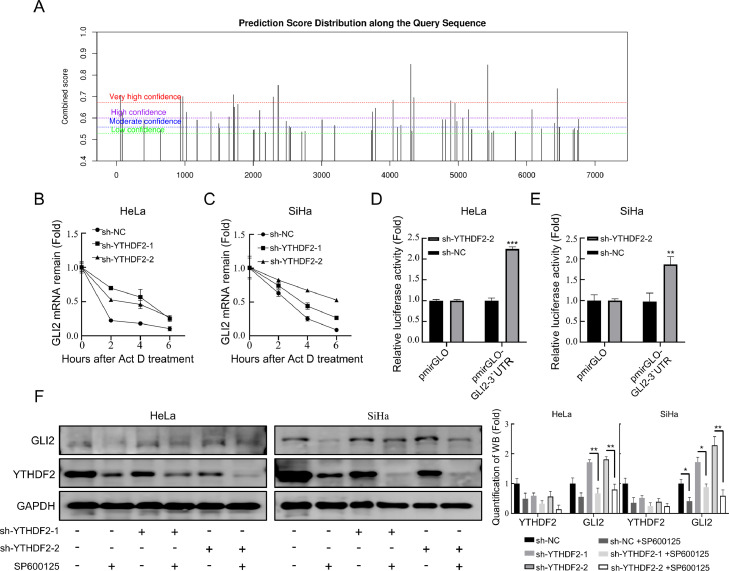
Knockdown of YTHDF2 upregulates GLI2 expression by extending the half-life of GLI2 mRNA and activating JNK. (A) The SRAMP database was used to predict the m6A site of GLI2 mRNA. (B, C) Half-life of GLI2 was measured in HeLa and SiHa with control and YTHDF2 knockdown groups using actinomycin D. (D, E) Dual fluorescein reporter gene assay to detect YTHDF2 binding to GLI2 3′UTR. (F) SP600125 (2 μM) was added to control and YTHDF2 stable knockdown HeLa and SiHa cells. Representative Western blots of YTHDF2 and GLI2 and their quantitative analysis in these cells after 24 h culturen. The data represent the mean ± SD of three independent experiments. ANOVA or *t*-tests were used for statistical analysis. * *p* < 0.05, ** *p* < 0.01, *** *p* < 0.001 indicate a significant difference between the indicated groups.

To determine the mechanism by which YTHDF2 knockdown up-regulated GLI2 expression, the presence of m6A sites on GLI2 mRNA, which can function as YTHDF2 binding sites, was analyzed. Prediction of the m6A site of GLI2 mRNA by the SRAMP database revealed that multiple m6A modifications may exist at the 3′ end of GLI2 (Fig. 8A). The half-life of the mRNA was then evaluated by transcriptional blockade with actinomycin D, and it was found that the knockdown of YTHDF2 prolonged the half-life of GLI2 mRNA (Figs. 8B and 8C). Subsequently, using a dual-luciferase reporter gene assay, it was found that the knockdown of YTHDF2 effectively increased luciferase expression (Figs. 8D and 8E). Previous reports suggested that JNK activation promotes GLI2 stability by inhibiting GLI2 ubiquitination [41,42]. The protein level of GLI2 was examined by introducing SP600125 into cervical cancer cells in which YTHDF2 was knocked down (Fig. 8F). It was found that the protein expression level of GLI2 was increased by the addition of SP600125, indicating that the activation of JNK could up-regulate the expression level of GLI2. These results suggest that knockdown of YTHDF2 can lead to the up-regulation of GLI2 expression by prolonging the mRNA half-life of GLI2 and increasing the expression level of GLI2 protein through JNK phosphorylation.

### Knockdown of YTHDF2 inhibits the progression of cervical cancer cell xenografts in nude mice

**Fig. 9:**
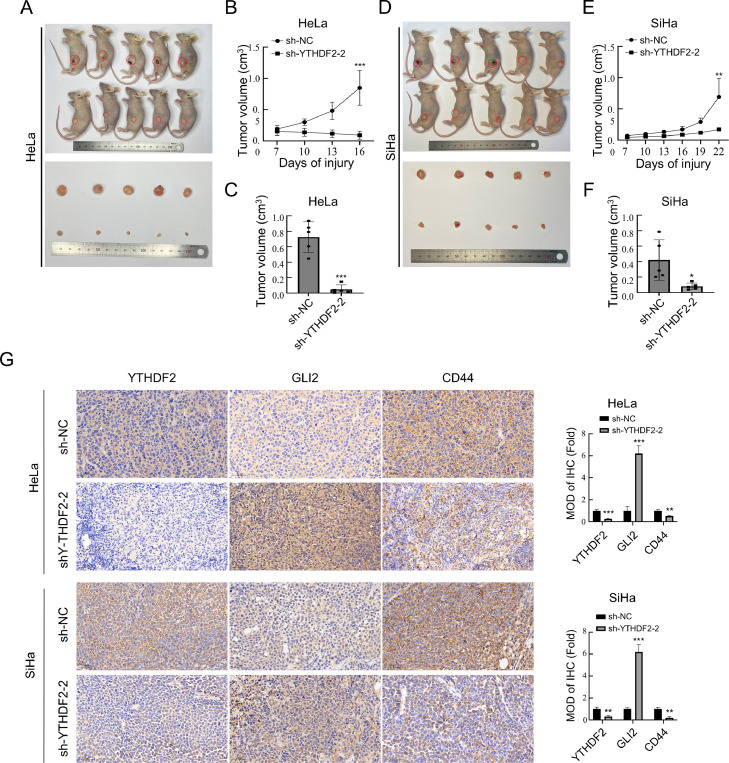
Knockdown of YTHDF2 inhibits the progression of cervical cancer cell xenografts in nude mice. (A) Xenograft tumor model was established in nude mice by subcutaneous injection of control and YTHDF2 knockdown groups of HeLa cells. (B) Xenograft tumor volume was measured during growth in nude mice after inoculation of control and YTHDF2 knockdown groups of HeLa cells. (C) Xenograft tumor volume was measured after sacrifice of nude mice in HeLa cell control and YTHDF2 knockdown groups. (D) Xenograft tumor model was established in nude mice by subcutaneous injection of control and YTHDF2 knockdown groups of SiHa cells. (E) Xenograft tumor volume was measured during growth in nude mice after inoculation of control and YTHDF2 knockdown groups of SiHa cells. (F) Xenograft tumor volume was measured in both SiHa cell control and YTHDF2 knockdown groups after nude mice were sacrificed. (G) Representative images of IHC staining showing YTHDF2, GLI2, and CD44 in two subcutaneous tumor models of HeLa and SiHa cells and their quantitative analysis. The data represent the mean ± SD of three independent experiments. ANOVA was used for statistical analysis. * *p* < 0.05, ** *p* < 0.01, *** *p* < 0.001 indicate a significant difference between the indicated groups.

To examine the impact of YTHDF2 knockdown on cervical cancer in vivo, a nude mouse xenograft tumor model of YTHDF2 knockdown and control cervical cancer cells was established. The volume of the xenograft tumors was monitored during their growth, and the volume of the tumors was measured after sacrificing the nude mice. Figs. 9A–9F present the outcomes of YTHDF2 knockdown in HeLa and SiHa cell lines, displaying xenograft tumor size, volume detection during growth, and final xenograft tumor volume at the time of sacrifice of nude mice. Xenograft tumors were fixed, sectioned, and subjected to immunohistochemistry, and the results are shown in Fig. 9G. These results indicated that knockdown of YTHDF2 inhibited the progression of cervical cancer cells in xenograft tumors in nude mice.

## Discussion

Cervical cancer ranks fourth among cancers commonly afflicting women, with significant lethality [1]. It exhibits a higher incidence and lethality in low- and middle-income countries than in developed countries [43,44]. HPV vaccines are effective in preventing most cases of cervical cancer [6]. However, vaccination may not be accessible to all, and some individuals may still develop cervical cancer despite receiving the vaccine [5]. Early detection of cervical cancer through reliable screening is crucial for successful treatment, which may include surgery, radiotherapy, and other traditional approaches with high success rates [9]. However, the efficacy of existing treatments for late-stage cervical cancer is inadequate. This study investigated the pathogenesis of cervical cancer, identified promising targets for cervical cancer diagnosis and gene therapy, and introduced novel concepts and treatment strategies for cervical cancer management.

YTHDF2 binds to m6A sites on RNA, reducing its stability [11]. In the study, the effects of YTHDF2 knockdown on cervical cancer cells were investigated. The results showed that the knockdown of YTHDF2 suppressed the progression of cervical cancer cells by inhibiting tumor stemness and promoting apoptosis. These results suggest that YTHDF2 knockdown leads to changes in the pathways regulating tumor stemness and apoptosis. However, no significant effect on cell viability was observed in cervical cancer cells overexpressing YTHDF2 compared with those with YTHDF2 knockdown. The results of functional enrichment indicate affected RNA and protein metabolic pathways, cellular stress granules, and enrichment for protein processing in endoplasmic processes. YTHDF2 can bind to the methylation site of the 3′ UTR of an mRNA and degrade the mRNA. This suggests that knockdown of YTHDF2 may lead to oxidative stress and ERS. ERS and UPR mediate several molecular and biochemical mechanisms that affect cell proliferation, differentiation, and death [28,29]. Previous studies have reported a relationship between ERS and the stemness of tumor cells [45,46]. Additionally, it has been suggested that knockdown of YTHDF2 in triple-negative breast cancer cells induces proteotoxicity through ERS [21]. It was later confirmed that the reduction of YTHDF2 boosted ROS production in cervical cancer cells and increased the production of ERS-linked proteins. The inclusion of 4PBA, an ERS-inhibitor, restored the expression of proteins that aid in tumor stemness and apoptosis. Additionally, the size of the cervical cancer cells increased when they were suspended in spheres following YTHDF2 knockdown. These results suggest that YTHDF2 knockdown promotes tumor stemness and inhibits apoptosis by promoting ERS. It has been reported that the JNK pathway is activated during ERS in cervical cancer [41,42]. Verification performed to determine whether there was corresponding activation of JNK, demonstrated that JNK was indeed phosphorylated after YTHDF2 knockdown. Additionally, the expression levels of ERS-related proteins were down-regulated after the introduction of the JNK inhibitor SP600125. This study indicates that knockdown of YTHDF2 in cervical cancer cells induces ERS through the phosphorylation of JNK.

**Fig. 10:**
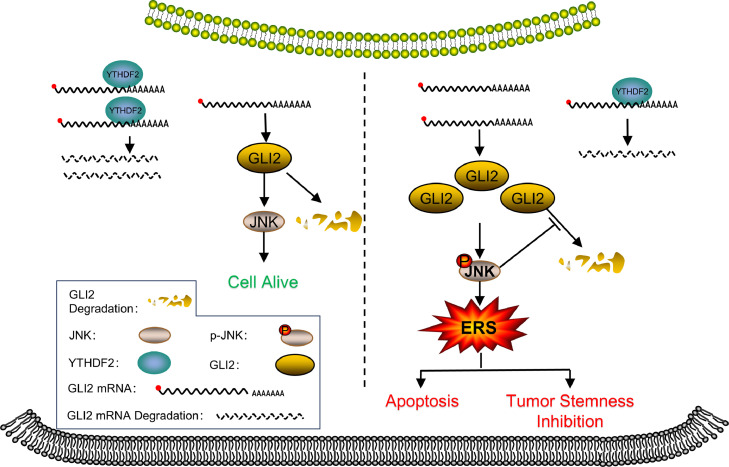
Schematic representation of the molecular mechanism through which YTHDF2 knockdown affects cervical cancer cells.

To investigate whether there is a target mRNA of YTHDF2 that plays an important role in the ERS that occurs after knockdown of YTHDF2, GLI2 was analyzed and verified as a possible target gene for the action of YTHDF2.The GLI family zinc finger 2 gene encodes a protein that belongs to the C2H2-type zinc finger protein subclass of the Gli family. Many studies have shown that GLI2 is involved in the development of cancer. Phosphorylation of JNK and promotion of ERS occur due to the up-regulation of GLI2 expression following the knockdown of YTHDF2 in cervical cancer cells. JNK inhibitors were applied to cervical cancer cells transiently expressing GLI2, and it was found that ERS occurs due to phosphorylation of JNK after the up-regulation of GLI2 expression. These findings suggest a causal connection between the up-regulation of GLI2 expression and the promotion of ERS through JNK phosphorylation. It was later discovered that YTHDF2 affects GLI2 expression. Analysis indicates a possible m6A site in the 3′ UTR of GLI2 mRNA. This was then verified, demonstrating the binding between YTHDF2 and the 3′ UTR of GLI2 mRNA. Consequently, the half-life of GLI2 was reduced. Additionally, activation of the JNK pathway reduces the ubiquitination levels of GLI2 proteins, thus preventing degradation. In this study, it was found that phosphorylation of JNK increases the expression of GLI2. Additionally, GLI2 up-regulation promotes JNK activation, which in turn stabilizes the GLI2 protein. These results were also observed in experiments using xenograft tumors in nude mice. These results suggest that the knockdown of YTHDF2 promotes ERS by phosphorylating JNK through increasing GLI2 expression, leading to the suppression of tumor stemness and apoptosis in cervical cancer cells (Fig. 10).

Taken together, high YTHDF2 expression may be necessary for maintaining tumor stemness in cervical cancer cells. Additionally, spherical cervical cancer cells in suspension culture medium promoted stemness gene expression and the up-regulation of YTHDF2. It is unclear whether the same phenomenon occurs when tumor cells enter the bloodstream and form spheres in vivo. The knockdown of YTHDF2 significantly impacts the viability of the suspended cells. It is worth exploring whether the delivery of siYTHDF2 RNA via a carrier molecule into the bloodstream can effectively eliminate tumor cells from entering the bloodstream to prevent the formation of metastatic foci during tumor treatment or surgical resection. These findings raise intriguing inquiries that warrant further investigation.

## CRediT authorship contribution statement

**Fujian Wan:** Writing – review & editing, Writing – original draft, Investigation, Data curation, Conceptualization. **Fengwu Qiu:** Writing – review & editing, Visualization, Supervision, Funding acquisition. **Yang Deng:** Writing – original draft, Methodology. **Hao Hu:** Writing – original draft, Methodology. **Yingjie Zhang:** Writing – original draft, Validation. **Jia-Yu Zhang:** Validation, Writing – original draft. **Pei Kuang:** Writing – original draft, Validation. **Haoyu Tian:** Writing – original draft. **Dewang Wu:** Writing – original draft, Software. **Hang Min:** Writing – original draft, Formal analysis. **Jiapeng Li:** Writing – review & editing. **Jing Xu:** Resources, Project administration, Funding acquisition, Writing – review & editing. **Jun Zhou:** Writing – review & editing, Writing – original draft, Supervision, Conceptualization.

## Declaration of competing interest

The authors declare no conflict of interest.

## Appendix Supplementary materials

Image, application 1
